# Comprehensive Plasma N-Glycoproteome Profiling Based on EThcD-sceHCD-MS/MS

**DOI:** 10.3389/fchem.2022.920009

**Published:** 2022-06-20

**Authors:** Yonghong Mao, Tao Su, Tianhai Lin, Hao Yang, Yang Zhao, Yong Zhang, Xinhua Dai

**Affiliations:** ^1^ Institute of Thoracic Oncology, West China Hospital, Sichuan University, Chengdu, China; ^2^ Institutes for Systems Genetics, West China Hospital, Sichuan University, Chengdu, China; ^3^ Department of Urology, Institute of Urology, West China Hospital, Sichuan University, Chengdu, China; ^4^ Mass Spectrometry Engineering Technology Research Center, Center for Advanced Measurement Science, National Institute of Metrology, Beijing, China

**Keywords:** mass spectrometry, plasma, N-glycoproteomics, combinatorial peptide ligand library, EThcD-sceHCD

## Abstract

Glycoproteins are involved in a variety of biological processes. More than one-third of the plasma protein biomarkers of tumors approved by the FDA are glycoproteins, and could improve the diagnostic specificity and/or sensitivity. Therefore, it is of great significance to perform the systematic characterization of plasma N-glycoproteome. In previous studies, we developed an integrated method based on the combinatorial peptide ligand library (CPLL) and stepped collision energy/higher energy collisional dissociation (sceHCD) for comprehensive plasma N-glycoproteome profiling. Recently, we presented a new fragmentation method, EThcD-sceHCD, which outperformed sceHCD in the accuracy of identification. Herein, we integrated the combinatorial peptide ligand library (CPLL) into EThcD-sceHCD and compared the performance of different mass spectrometry dissociation methods (EThcD-sceHCD, EThcD, and sceHCD) in the intact N-glycopeptide analysis of prostate cancer plasma. The results illustrated that EThcD-sceHCD was better than EThcD and sceHCD in the number of identified intact N-glycopeptides (two-folds). A combination of sceHCD and EThcD-sceHCD methods can cover almost all glycoproteins (96.4%) and intact N-glycopeptides (93.6%), indicating good complementarity between the two. Our study has great potential for medium- and low-abundance plasma glycoprotein biomarker discovery.

## 1 Introduction

Human plasma is a critical area of clinical and fundamental research, as it contains a large number of disease candidate biomarkers (Jacobs et al., 2005). Protein biomarkers in the plasma change in concentration or state associated with a biological status or disease, offering great potential for patient diagnosis, risk stratification, and disease prevention (Moremen et al., 2012; Silsirivanit, 2019). More than one-third of plasma tumor protein biomarkers approved by the FDA are glycoproteins (Silsirivanit, 2019). Glycosylation has been recognized as one of the most multifunctional protein modifications (Moremen et al., 2012). It plays a vital role in the progression of various cancers (Ohtsubo and Marth, 2006). However, the dynamic range of plasma proteins can exceed 10^9^, and many potential biomarkers are low-abundance proteins (Jacobs et al., 2005). Moreover, glycoproteomic analysis is difficult due to the microheterogeneity and macroheterogeneity of glycosylation, and other special properties (Pujic and Perreault, 2021). Hence, comprehensive identification of human plasma glycoproteome (including intact glycopeptides, glycoproteins, glycosites, and glycans) is an important way to discover new biomarkers.

In the past few years, some researchers have made contributions to study plasma proteome and glycoproteome. In 2005, the Human Plasma Proteome Project identified a total of 7518 proteins and isoforms in plasma (Muthusamy et al., 2005). As a result of improvements in proteomic technologies, 10,546 plasma proteins were included in this database in 2014 (Nanjappa et al., 2014). However, the database of human plasma glycoproteome is still immature. This may be due to difficulties in sample processing, mass spectrometry analysis, and data processing. Still, experts in the field are doing their best to overcome these difficulties. For example, many enrichment materials or methods were developed to remove non-glycoproteins or non-glycopeptides in plasma (Palmisano et al., 2010; Sun et al., 2017; Yang et al., 2017; Zhang et al., 2017; Wu et al., 2018; Chen et al., 2019; Sun et al., 2019; Zhang et al., 2019; Zhang et al., 2020a). Moreover, in order to deplete high-abundance proteins, immunodepletion technologies and ProteoMiner protein enrichment methods were used to remove the abundant plasma proteins (Luque-Garcia and Neubert, 2007; Sennels et al., 2007).

In recent years, various fragmentation techniques (EThcD, sceHCD, EThcd-sceHCD, etc.) have appeared as valuable approaches for plasma glycoproteomics. EThcD means ETD followed by supplemental HCD, which fragments parent ions via ETD first and then the products ions are fragmented via HCD (Zhang et al., 2018), while sceHCD means stepped collision energy HCD (beam-type collisional activation) on Orbitrap systems (Riley et al., 2020). Many intact glycopeptide search engines, such as MSFragger-Glyco, Byonic, pGlyco, Glyco-Decipher, and StrucGP, have improved the accuracy of intact glycopeptide identification (Bern et al., 2012; Liu et al., 2017; Lu et al., 2020; Polasky et al., 2020; Shen et al., 2021; Fang et al., 2022). Kawahara et al. evaluated several search strategies and provided valuable information for serum or plasma glycoproteomic studies (Kawahara et al., 2021).

In previous studies, we developed Glyco-CPLL for human plasma N-glycoproteome profiling based on sceHCD and established a large database (Zhang et al., 2020b). CPLL means combinatorial peptide ligand library (a diverse library of hexapeptides that act as binders for proteins), which is a plasma sample preparation tool used for the compression of the dynamic range of the protein concentration and maintaining representatives of all proteins. When plasma samples were applied to the CPLL beads, the high-abundance proteins saturated their high-affinity ligands, and excess proteins were washed away. In contrast, the medium- and low-abundance proteins were concentrated on their specific affinity ligands (Sennels et al., 2007). Recently, we integrated EThcD and sceHCD into a glycoproteomic workflow (Zhang et al., 2021a; Zhang et al., 2021b). The results clearly showed that EThcD-sceHCD can improve the intact glycopeptide analysis performance of HIV-1 gp120, IgG subclasses, and complex clinical samples (Zhang et al., 2021a; Zhang et al., 2021b; Zeng et al., 2022).

Herein, we aim to improve the accuracy and depth of human plasma intact glycopeptide identification based on the new analysis method. More precisely, we integrated Glyco-CPLL into EThcD-sceHCD, compared the performance of different dissociation methods (EThcD-sceHCD, EThcD, and sceHCD), and determined its superiority for plasma N-glycoproteomic studies.

## 2 Experimental Section

### 2.1 Materials

Chemical reagents, such as dithiothreitol (DTT), iodoacetamide (IAA), formic acid (FA), trifluoroacetic acid (TFA), Tris base, and urea, were purchased from Sigma (St. Louis, MO, United States). Acetonitrile (ACN), ethanol (EtOH), methanol (MeOH), and acetic acid (HAc) were purchased from Merck (Darmstadt, Germany). Sequencing grade trypsin and Lys-C were obtained from Promega (Madison, WI, United States). ProteoMiner column was purchased from Bio-Rad Laboratories (Hercules, CA). Zwitterionic HILIC (Zic-HILIC) was purchased from Fresh Bioscience (Shanghai, China). The C8 extraction disks were purchased from 3M Empore (St. Paul, MN, United States). All other materials were purchased from Sigma-Aldrich or Thermo Fisher Scientific.

### 2.2 Biospecimen Collection

Diagnosis and confirmation of PCa patients were performed in the Department of Urology, West China Hospital of Sichuan University, Chengdu, China. Blood was collected into EDTA anticoagulant tubes. After centrifuging at 300 g for 10 min at 4°C, plasma was collected and stored at −80°C. Written informed consents were collected. The experiment was performed in accordance with the guidelines of the Chinese Medical Ethics Committee. The experiment was approved by the Ethics Committee at West China Hospital.

### 2.3 Sample Pretreatment

Plasma samples from PCa patients were pooled before analysis. The plasma proteins were prepared using the CPLL method as described before. Specifically, 200 μl pooled plasma was loaded into the ProteoMiner column and incubated at room temperature for 2 h. After centrifuging at 1,000 g for 30 s at 4°C, the column was washed with 200 μl of PBS and ddH_2_O, and the CPLL bound proteins were eluted with 20 μl of elution buffer (8 M urea, 2% CHAPS).

### 2.4 Reduction, Alkylation, and Digestion

The eluent was proteolyzed following the filter-aided sample preparation (FASP) protocol. Briefly, after diluting 20 times with UA solution (8 M urea in 0.1 M Tris-HCl, pH 8.5), the eluent was added to a 30-kDa filter. After carrying out reduction reaction by adding 20 mM DTT for 4 h at 37°C, alkylation reaction was carried out by adding 50 mM IAA and incubating the mixture in the dark for 1 h. Proteins were digested by adding trypsin/Lys-C (1:50) to each filter tube. The peptides were collected by washing three times with 100 μl of water.

### 2.5 Intact Glycopeptide Enrichment

Intact N-glycopeptides were enriched using Zic-HILIC materials. Specifically, 200 μg of tryptic peptides and 10 mg of Zic-HILIC materials were mixed in 70% ACN/0.2% TFA solution. Then, the mixture was transferred to a pipette tip packed with a C8 membrane. Hydrophobic peptides were washed with 70% ACN/0.2% TFA, and intact N*-*glycopeptides were eluted with 70 μl of 0.1% TFA and collected in a 1.5-ml tube. The eluent was dried using a SpeedVac for further analysis.

### 2.6 LC-MS/MS Analysis

The dried intact N-glycopeptides were resuspended in 20 μL of 0.1% FA individually. Then 5 μL of samples was taken for analysis on an Orbitrap Fusion Lumos mass spectrometer (Thermo Fisher, United States). All intact N-glycopeptides were separated on a column (ReproSil-Pur C18-AQ, 1.9 μm, 75 μm inner diameter, length 20 cm; Dr Maisch) over a 78-min gradient at a flow rate of 300 nL/min. Three different fragmentation modes (EThcD, sceHCD, and EThcD-sceHCD) were used for intact N-glycopeptide analysis.

For EThcD-MS/MS and sceHCD-MS/MS, the parameters were as follows: MS1 was analyzed in the range of 800–2000 m*/z* at an Orbitrap resolution of 60,000. The RF lens, AGC target, MIT, exclusion duration, and cycle time were 40%, custom, 50 ms, 15 s, and 3 s, respectively. The precursor ion in MS2 experiment was performed at 2 m*/z* and acquired at an Orbitrap resolution of 30,000. The AGC target and MIT were custom and 150 ms, respectively. EThcD collision energy was 35%, while the sceHCD mode was turned on with an energy difference of ±10% (20-30-40%).

For EThcD-sceHCD-MS/MS, the analysis was performed using an alternative fragmentation between the EThcD and sceHCD modes in a duty cycle. In the EThcD duty cycle, MS1 was analyzed in the range 800–2000 m*/z* at an Orbitrap resolution of 60,000. The RF lens, AGC target, MIT, and exclusion duration were 40%, 2.0 e^5^, 50 ms, and 15 s, respectively. MS2 was analyzed at 2 m*/z* at an Orbitrap resolution of 30,000. The AGC target, MIT, and EThcD type were standard, 150 ms, and 35%, respectively. In the sceHCD duty cycle, MS1 was analyzed in the range 800–2000 m*/z* at an Orbitrap resolution of 60,000. The RF lens, AGC target, MIT, exclusion duration, and cycle time were 40%, standard, auto, 15 s, and 1 s, respectively. The precursor ion in the MS2 experiment was selected at 1.6 m*/z* and acquired at an Orbitrap resolution of 30,000. The AGC target, MIT, and HCD collision energy were 200%, auto, and 30%, respectively. Moreover, the sceHCD mode was turned on with an energy difference of ±10% (20-30-40%). Although the data-dependent mode cycle time of each method was set as 3 s, there were differences in scanning speed between different modes (ETD has a slower scan speed). Therefore, the number of precursors selected for MS/MS for each method may be different.

### 2.7 Data Analysis

The data files were searched against the Human UniProt database (version 2015_03, 20,410 entries) using Byonic software (version 3.10.10, Protein Metrics, Inc.). Mass tolerance for precursors and fragment ions were set as ± 6 ppm and ± 20 ppm, respectively. Two missed cleavage sites were allowed. Carbamidomethyl (C) was set as fixed modification. Variable modifications contained oxidation (M) and acetyl (protein N-term). Additionally, the “182 human N-glycans” was set as the N-glycan modification. Protein groups were filtered to 1% FDR. Quality control methods for intact N-glycopeptide identification included a Byonic score of over 200, a logProb value of over 2, and at least five amino acids. ANOVA was used for statistical comparison among three groups, and Student’s *t*-test was used for statistical comparison between two groups (SPSS Statistics 19.0). The homogeneity test was performed. The error bar denotes SD. *p*-value < 0.01 was considered significant. The raw data can be obtained *via* ProteomeXchange with identifier PXD030622.

## 3 Results and Discussion

**FIGURE 1:**
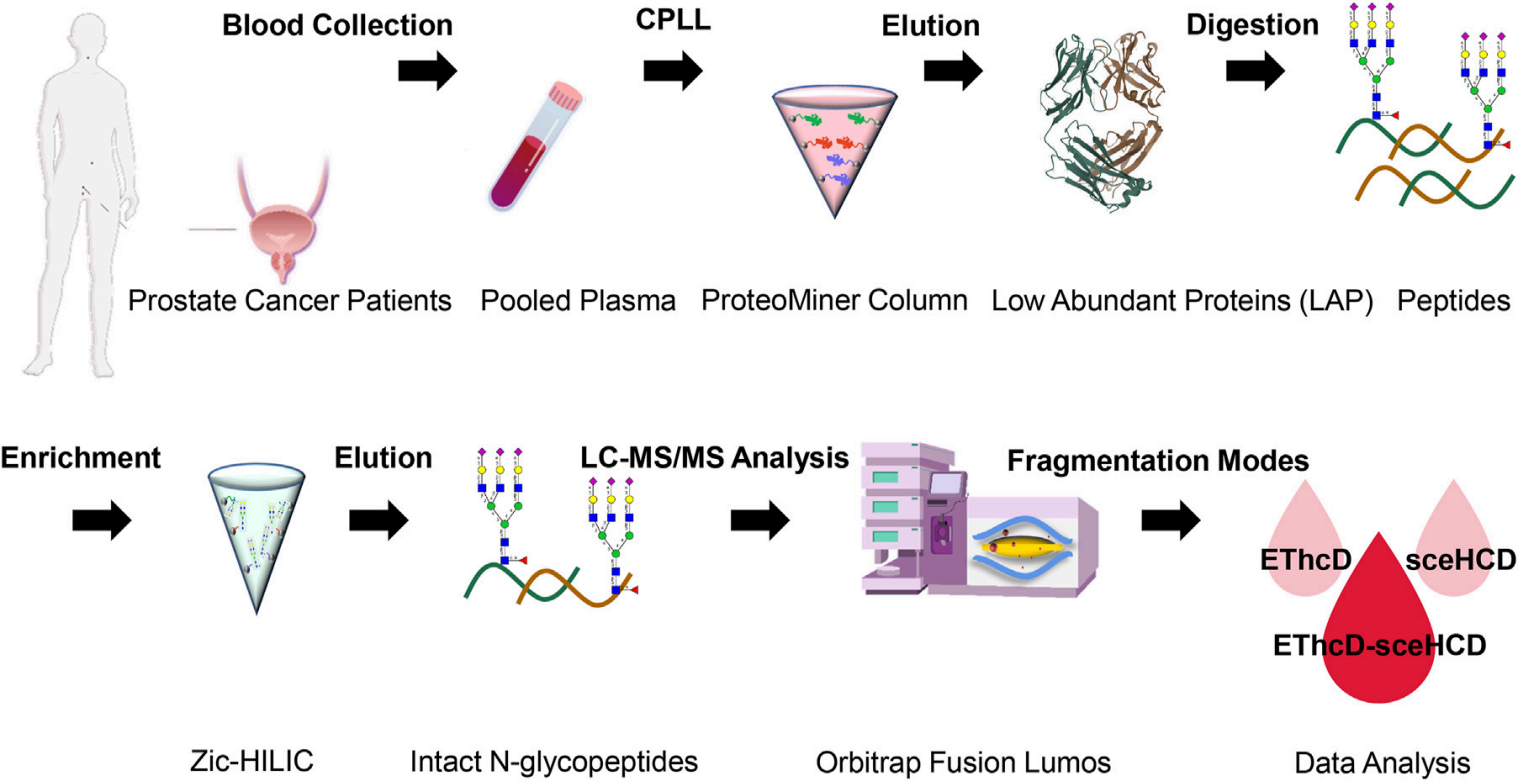
Schematic representation of the workflow for human plasma LAP intact N-glycopeptide analysis using different dissociation methods (EThcD, sceHCD, and EThcD-sceHCD).

Recently, we proved that EThcD-sceHCD has better performance in the intact glycopeptide analysis of HIV-1 gp120 and IgG subclasses (Zhang et al., 2021a; Zhang et al., 2021b). However, whether this method can be applied to complex plasma samples is unknown. To evaluate the efficiency of the method on human plasma samples and systematically characterize plasma N-glycoproteome in PCa patients, the following experiment was designed (Figure 1). In detail, low-abundance proteins (LAPs) were extracted from pooled PCa patients’ plasma using the ProteoMiner protein enrichment system which contains the combinatorial peptide ligand library (CPLL) (Bandow, 2010; Moggridge et al., 2019; Zhang et al., 2020b; Palstrøm et al., 2020). After digestion by trypsin and Lys-C, Zic-HILIC materials were used to enriched intact N-glycopeptides (Di Palma et al., 2011). Then, the same number of samples were analyzed using three fragmentation methods (EThcD, sceHCD, and EThcD-sceHCD) (Figure 1). All raw data files were searched using Byonic software. The search results were analyzed statistically and compared systematically (Supplementary Table S1).

**FIGURE 2:**
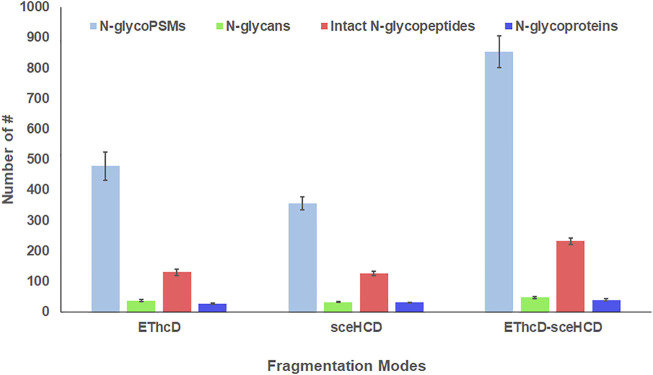
Comparison of the number of N-glycoPSMs, N-glycans, intact N-glycopeptides, and N-glycoproteins from pooled PCa patients’ plasma using different dissociation methods. (ANOVA was used for the statistical comparison among three groups, and Student’s *t*-test was used for the statistical comparison between two groups. The homogeneity test was performed. The error bar denotes SD.)

**FIGURE 3:**
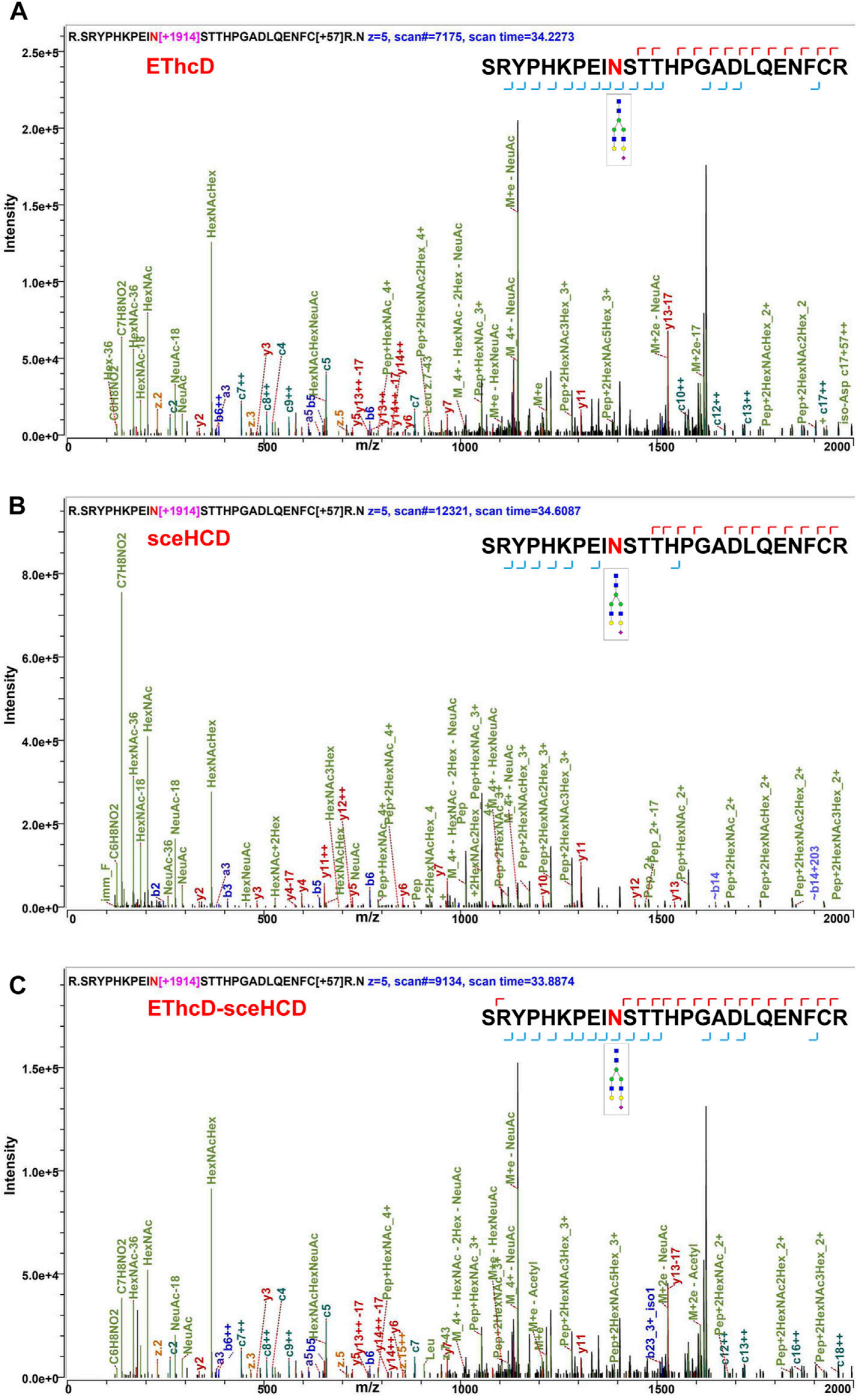
Comparison of the EThcD **(A)**, secHCD **(B)**, and EThcD-sceHCD **(C)** spectra of prothrombin intact N-glycopeptides (N143) from human plasma.

Using the same quality control standards as described earlier, the identified N-glycoPSMs, N-glycans, intact N-glycopeptides, and N-glycoproteins from pooled PCa patients’ plasma were analyzed and compared. EThcD-sceHCD outperformed EThcD and sceHCD in every number of identification (*p* < 0.001, ANOVA) (Figure 2). In particular, EThcD-sceHCD identified nearly twice (232) as many intact N-glycopeptides as EThcD (129) and sceHCD (126) (Figure 2). It has been reported that sceHCD can provide more information on fragment ions than other modes (Riley et al., 2020; Klein and Zaia, 2020). However, it cannot provide the accurate glycosite location and glycan composition information when one sequence contains more than one glycosite (Zhang et al., 2021b). EThcD can produce a greater proportion of fragment ions via both ETD and HCD, and thereby provide more key information for the unambiguous identification of both glycosites and glycans (Yu et al., 2017; Saba et al., 2012; Ma et al., 2016). Nevertheless, ETD has limited dissociation efficiency. Hence, we proposed EThcD-sceHCD, which alternatively fragment samples between EThcD and sceHCD modes in a duty cycle (Zhang et al., 2021b). That is, EThcD-sceHCD has better spectra quality and higher dissociation efficiency. For example, the three methods can provide abundant information about N-glycosite (N143) localization and the N-glycan composition (HexNAc(4)Hex (5)NeuAc (1)) of one N-glycopeptide from prothrombin (Figure 3). Both EThcD and EThcD-sceHCD can provide complex and informative fragment ions (glycan fragments, b/y/c/z ions, and Y ions) (Figures 3A, C). However, sceHCD provided less information (glycan fragments, b/y ions, and Y ions) (Figure 3B). In other words, EThcD and EThcD-sceHCD can provide more accurate N-glycosylation modification information than sceHCD, although they have lower intensity than sceHCD because ETD has limited scanning speed (Figure 3). In addition, we analyzed the largest Byonic score distribution of intact N-glycopeptide spectra from different fragmentation modes (Supplementary Table S2). Byonic score is the “raw” indicator of PSM correctness, reflecting the absolute quality of the PSM (Bern et al., 2012). As expected, the largest Byonic scores of the 69.8% (252/361) intact N-glycopeptides were obtained by EThcD-sceHCD, and the other 17.5% (63/361) and 12.7% (46/361) intact N-glycopeptides were obtained from EThcD and sceHCD, respectively (Supplementary Table S2). The results showed that EThcD-sceHCD can fragment peptide backbone more extensively than EThcD and sceHCD. The reason may be that different peptide backbones obtained better fragmentation ions under different fragmentation modes. Bertozzi et al. compared multiple mass spectrometry dissociation methods and concluded that sceHCD significantly outperformed EThcD for N-glycopeptide identification from HEK293 whole cell lysates (Riley et al., 2020). Our results support this conclusion (Zeng et al., 2022). However, sceHCD outperformed the EThcD-sceHCD method for N-glycopeptide identifications from the urine of IgAN patients, HepG2 cells, and thyroid cancer tissues. And the interesting thing is that EThcD-sceHCD outperformed the sceHCD method for N-glycopeptide identification from plasma treated with/without CPLL. All of these revealed that EThcD-sceHCD can outperform EThcD and sceHCD in the number of identified intact N-glycopeptides and accuracy of plasma intact N-glycopeptide identification.

**FIGURE 4:**
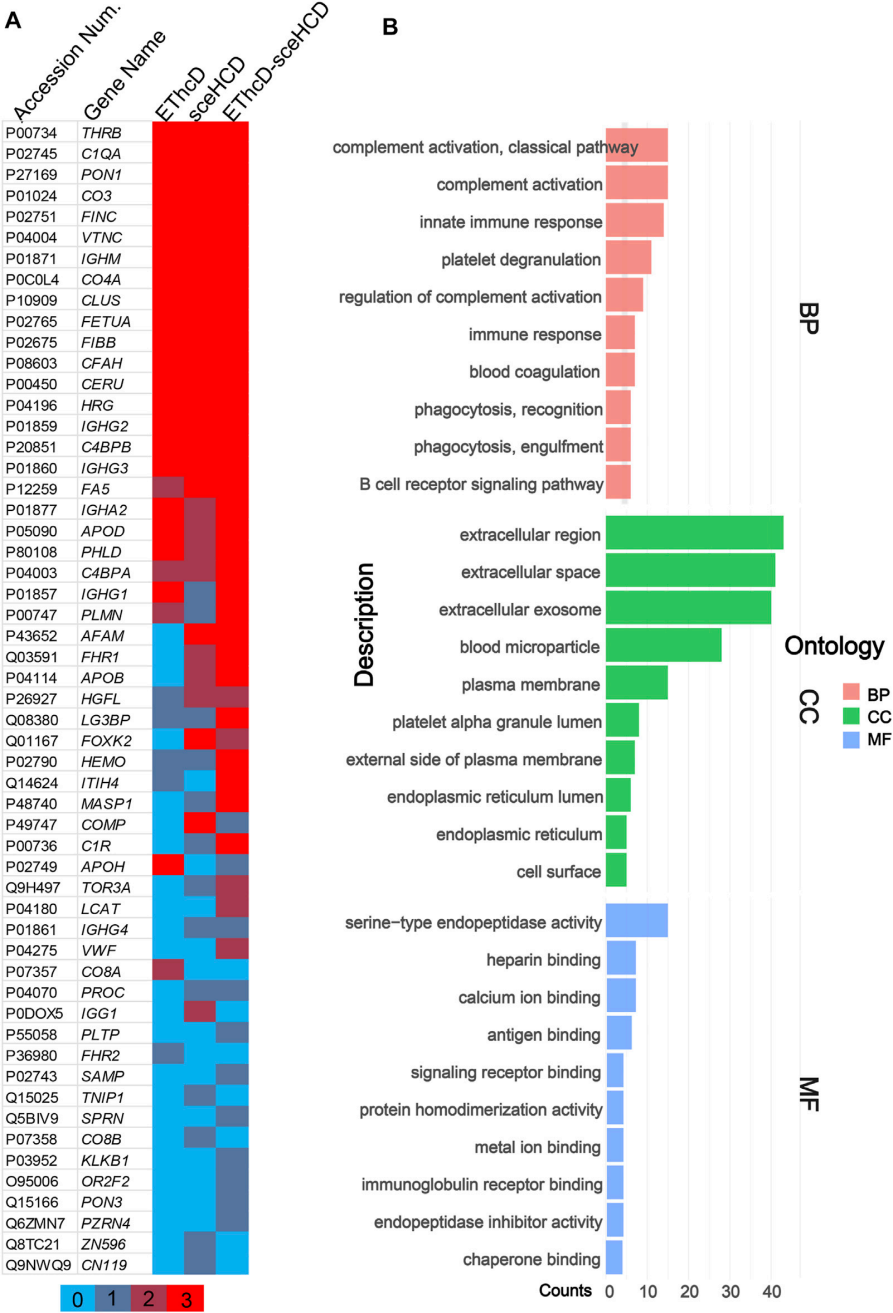
Global analysis of identified plasma N-glycoproteins. **(A)** Heat map of identified plasma N-glycoproteins using different fragmentation modes. Colored lines indicate the number of repetitions identified. **(B)** Gene ontology (GO) biological process (BP), cellular component (CC), and molecular function (MF) enrichment analysis.

Meanwhile, we analyzed the identified plasma N-glycoproteins (Figure 4). EThcD-sceHCD identified most N-glycoproteins (48/56), and 77.1% N-glycoproteins can be identified at least twice. This result is obviously better than that obtained using EThcD (26/56) and sceHCD (29/56). Integrating sceHCD and EThcD-sceHCD, 96.4% (54/56) N-glycoproteins can be identified (Figure 4A). Moreover, using the Wukong platform, gene ontology (GO) enrichment analysis was performed to determine the biological process (BP), cellular component (CC), and molecular function (MF) of these plasma glycoproteins in PCa patients. As shown in Figure 4B, they were involved in many biological processes, such as complement activation and immune response, mainly localized in the extracellular space. The major molecular functions were catalytic activities and binding (Figure 4B). These results suggested that the function of N-glycoproteins in PCa patients’ circulatory system may be dysfunctional (Zhang et al., 2020b).

**FIGURE 5:**
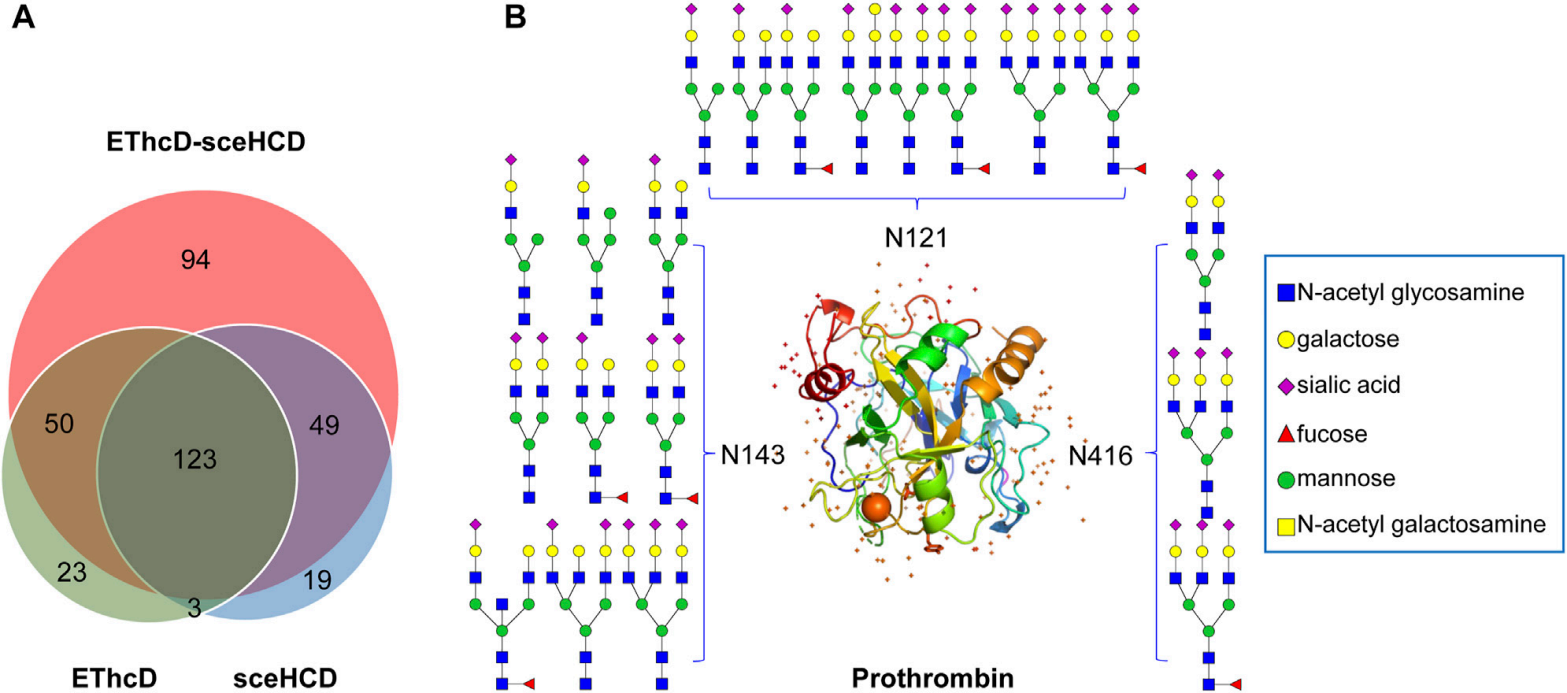
Global analysis of identified plasma intact N-glycopeptides. **(A)** Comparison of identified intact N-glycopeptides using EThcD, secHCD, and EThcD-sceHCD. **(B)** N-glycosites (N121, N143, and N416) and deduced N-glycans were demonstrated in the three-dimensional structure of the prothrombin (PDB code: 1A2C) from pooled PCa patients’ plasma.

Based on the comprehensive PCa patients’ plasma N-glycoproteomic information obtained in this work, we further compared these intact N-glycopeptides. EThcD-sceHCD can identify an additional 94 intact N-glycopeptides, while EThcD and sceHCD can identify only 23 and 19 intact N-glycopeptides, respectively (Figure 5A). Hence, using different fragmentation modes to analyze the same sample would produce complementary results. Based on these results, a PCa patient plasma N-glycoprotein database was established (Supplementary Table S1). For example, we found that prothrombin in PCa patients’ plasma is a completely sialylated glycoprotein, which plays a key role in blood homeostasis, wound healing, and inflammation. All of its reported N-glycosites (N121, N143, and N416) were identified in this work. The three N-glycosites were occupied by different amounts and types of N-glycans (Figure 5B). These results implied that our methods can decipher the site-specific glycosylation of human plasma glycoproteins. It is worth noting that we did not include healthy controls in this study because we were evaluating the usability and superiority of the EThcD-sceHCD technique in this work. We will apply this technique to a large cohort of clinical samples in our future research.

## 4 Conclusion

Herein, we once again proved the reliability and superiority of EThcD-sceHCD. By integrating CPLL into EThcD-sceHCD, we systematically compared the performance of different fragment methods in human plasma intact N-glycopeptide analysis. EThcD-sceHCD performed better in the accuracy and depth of intact N-glycopeptide identification. This finding would drive clinical plasma N-glycoproteomic methodological development and promote related application research.

## Data Availability

The datasets presented in this study can be found in online repositories. The names of the repository/repositories and accession number(s) can be found in the article/Supplementary Material.
